# Clinicopathological features of HER2 positive metastatic colorectal cancer and survival analysis of anti-HER2 treatment

**DOI:** 10.1186/s12885-022-09447-x

**Published:** 2022-04-01

**Authors:** Liu Yang, Wenfei Li, Zhihao Lu, Ming Lu, Jun Zhou, Zhi Peng, Xiaotian Zhang, Xicheng Wang, Lin Shen, Jian Li

**Affiliations:** 1 Department of Gastrointestinal Oncology, Key Laboratory of Carcinogenesis and Translational Research (Ministry of Education), Peking University Cancer Hospital & Institute, Fu-Cheng Road 52, Hai-Dian District, Beijing, 100142 China; 2 Department of oncology, People’s Hospital of Zhongmu, Zhengzhou, 451450 Henan Province China

**Keywords:** HER2 positive, Colorectal cancer, Trastuzumab

## Abstract

**Background:**

We aimed to investigate response and prognostic factors in patients with human epidermal growth factor receptor 2 (HER2) positive metastatic colorectal cancer (mCRC) and compare the curative effect on patients who received different therapy regimens (including chemotherapy and chemotherapy combined with targeted drugs).

**Methods:**

We retrospectively analyzed all HER2 positive mCRC patients treated at Peking University Cancer Hospital between September 2011 and February 2021. We divided 63 HER2 positive mCRC into group A and group B according to the use of trastuzumab or not. Besides, we assigned four subgroups according to the first-line therapies of *KRAS/NRAS/BRAF* WT patients. The Kaplan–Meier estimator was used to calculate PFS and OS. Univariable analysis and Cox proportional hazards models were used to analyze the association between clinicopathological features and survival outcomes.

**Results:**

Among 63 patients, 54 (85.7%) were *KRAS/NRAS/BRAF* wild-type (WT). Univariate analysis showed that the male sex, primary lesions in the right colon, simultaneous metastasis, and unresectable primary lesions were significant risk factors for poor survival of HER2 positive mCRC (*P* < 0.05). Using Cox proportional hazards models, we found that the two factors of gender and resection of primary lesions were independent prognostic factors *(P <* 0.05). The median PFS and median OS of HER2-positive patients with mCRC who received first-line treatment were 8.4 months [95% confidence interval (CI): 5.0–11.7] and 48.2 months (95% CI: 23.5–72.8), respectively. The log-rank test revealed a significant difference in median OS survival between group A and group B (χ^2^ = 5.852, *P* = 0.016), and the two groups were divided according to the use or absence of trastuzumab treatment. In *KRAS/NRAS/BRAF* WT patients, there was a significant difference in median PFS and median OS between the fourth group patients (chemotherapy plus trastuzumab) and each of the other three groups (*P* < 0.05).

**Conclusions:**

The two factors of gender and resection for primary lesion may be independent prognostic factors of advanced HER2 positive colorectal cancer patients. For patients with HER2-positive mCRC, patients in the chemotherapy combined with trastuzumab group have better efficacy than those without trastuzumab.

## Introduction

According to a status report on the cancer burden worldwide in 2018, colorectal cancer (CRC) ranks third in incidence and second in mortality [1]. About 30–40% of CRC patients had metastatic disease at the time of initial presentation [2, 3]. A subset of mCRC driven by amplification or mutation of HER2 was detected using functional and genomic analyses of patient-derived xenografts. Amplification of HER2 only accounts for approximately 3% of CRC patients [4, 5], increasing up to 5% in *KRAS* exon 2 WT tumors and 5.2% in *KRAS/BRAF* WT patients at stage IV [6, 7]. As the success of targeted therapies fostered the development of specific and effective therapeutics for colorectal malignancies, we should pay more attention to CRC patients with rare mutations. Activation of HER2 is regulated through the heterodimerization with diverse ligand-bound receptors, and previous clinical studies suggest that HER2 and EGFR have causative and progressive roles in tumor development [8]. The antibody trastuzumab was developed as a means of blocking HER2 [9]. Trastuzumab can improve overall survival and progression-free survival in HER2-positive women with metastatic breast cancer. While the relevance of the HER2 therapeutic target has been established, its role as a biomarker for prognosis in CRC remains uncertain. Thus, the role of anti-HER2 targeted therapy in mCRC with HER2 overexpression needs further investigation. Herein, we described the clinicopathological characteristics and treatment outcomes of HER2-positive mCRC and evaluated which patient or tumor-related factors are associated with patient outcomes in a real-life setting.

## Materials and methods

### Study population

We retrospectively counted 2460 mCRC patients treated at Peking University Cancer Hospital from 01 September 2011 to 19 February 2021, of which 63 were HER2 positive patients. The study was approved by the Institutional Ethics Committee of Peking University Cancer Hospital and signed informed consent was obtained from each participant.

The inclusion criteria for patients were as follows: 1) diagnosis of rectal or colon adenocarcinoma; 2) tumors must be HER2-positive; 3) at least one measurable target lesion as defined by Response Evaluation Criteria in Solid Tumors (RECIST); 4) good physical condition with an Eastern Cooperative Oncology Group score of 0 to 2; 5) no diseases of the heart, lung, liver, kidney, and no serious systemic diseases; 6) completed at least two cycles of treatment, including chemotherapy alone, trastuzumab combined with chemotherapy, bevacizumab combined with chemotherapy, or cetuximab combined with chemotherapy; 7) *KRAS*/*NRAS*/*BRAF* genes was detected in pathological samples of primary tumors or metastatic tumors.

HER2 positivity was defined as tumors with a HER2 score of 3+ in ≥50% of cells by immunohistochemistry (IHC), or a HER2 score of 2+ and a HER2:CEP17 ratio ≥ 2 in ≥50% of cells as determined by Fluorescence in Situ Hybridization (FISH). The molecular biomarkers included in the study were the *KRAS*, *NRAS* (exons 2, 3, and 4), and *BRAF* (V600E mutation allele) genes.

### Study design

We divided 63 HER2 positive mCRC into group A and group B according to the use of trastuzumab or not. Group A used trastuzumab, while group B did not use trastuzumab. We further divided group A patients into four subgroups, the first-, second-, third-, and fourth-line treatments according to the application time of trastuzumab.

Additionally, we evaluated four subgroups of *KRAS*/*NRAS*/*BRAF* WT patients according to four different first-line therapies: group 1 for chemotherapy alone, group 2 for chemotherapy plus bevacizumab (anti-VEGF), group 3 for chemotherapy plus cetuximab (anti-EGFR), and group 4 for chemotherapy plus trastuzumab (anti-HER2).

### Summary of treatments

In this study, patients mainly used single-agent chemotherapy, two-drug combination chemotherapy, and three-drug chemotherapy in each line of treatment. Some patients also used targeted drugs. The single-drug regimen mainly included capecitabine, tegafur, and raltitrexed. The two-drug combination chemotherapy regimens included irinotecan or oxaliplatin-based regimens, such as FOLFIRI, XELIRI, FOLFOX, and XELOX. The three-drug regimen included FOLFOXIRI and XELOXIRI. Targeted drugs mainly include trastuzumab, bevacizumab, and cetuximab.

### Evaluation of treatment response

The tumor response was assessed according to Response Evaluation Criteria in Solid Tumors (RECIST, Version 1.1).

### Statistical analysis

All data were analyzed using SPSS 26.0 software. Univariate analysis was performed using the log**-**rank test. Survival curves were generated using Kaplan-Meier estimates, differences between the curves were analyzed by log-rank tests. Multivariable Cox regression models were built for analysis of risk factors for survival outcomes. Statistical significance was set at a two-sided level of *P* < 0.05.

## Results

### Clinicopathological features and treatment response of HER2-positive mCRC

Sixty-three well-documented HER2-positive patients with mCRC were included in this study. The median patient age was 54 years (range, 27–78 yr). The median follow-up period was 23.7 (IQR 2.3–63.5) months. Patient pathological features are summarized in Table 1. The chemotherapy regimens in combination with the HER2-targeted treatment are summarized in Table 2. The chemotherapy regimens in *KRAS*/*NRAS*/*BRAF* WT patients of first-line therapies are summarized in Table 3. Treatment response evaluations in patients who received chemotherapy plus trastuzumab are summarized in Table 4.

**Table 1:** Characteristics of 63 HER2-positive mCRC

Characteristic	n (%)		n (%)
Age, Years		Tumor Differentiation	
< 54	30 (47.6)	Poor	7 (11.1)
≥ 54	33 (52.4)	Medium	54 (85.7)
Sex		Well	2 (3.2)
Male	41 (65.1)	primary lesion resection	
Female	22 (34.9)	Yes	52 (82.5)
Primary site		No	11 (17.5)
Left semi-colon and rectum	56 (88.9)	Metastasectomy	
Right colon	7 (11.1)	Yes	19 (30.2)
Number of metastatic organs		No	44 (69.8)
single/ (liver metastatic)	22/(18)(34.9)	Status of *KRAS NRAS BRAF*	
≥ 2/ (liver metastatic)	41/(15)(65.1)	Mutant	9 (14.3)
Metastatic patterns		WT	54 (85.7)
synchronous	36 (57.1)	Adjuvant chemotherapy	
Metachronous	27 (42.9)	Yes	25 (39.7)
Therapy Line		No	38 (60.3)
First-line	22 (34.9)	local therapies	
Second-Line	14 (22.2)	Yes	23 (36.5)
Third- or later-line	27 (42.9)	No	40 (63.5)

**Table 2:** Summary of chemotherapy regimens in combination with the HER2-targeted drugs

Group (A)	first-line	*N* = 3	second-line	*N* = 16	third-line	*N* = 9	four-line	*N* = 5
Combined treatment	CPT-11	2	CPT-11	13	CPT-11	7	CPT-11	3
	S1	1	XELOX	1	XELOX	1	CPT-11 + S1	1
			XELIRI	1	Xeloda	1	SOX	1
			Xeloda	1				

*CPT-11* Irinotecan, *S1* Tegafur, *XELOX* capecitabine plus oxaliplatin, *XELIRI* capecitabine plus irinotecan, *SOX* Tegafur plus oxaliplatin

**Table 3:** Summary of regimens in *63* patients of first-line therapies

WT (Group 1–4)	chemotherapy alone	*N* = 31	Plus Bev	*N* = 12	Plus CET	*N* = 8	Plus T-mab	*N* = 3
Combined treatment	XELIRI	2	FOLFIRI	1	XELIRI	1	CPT-11	2
	FOLFOXIRI	2	FOLFOX	1	FOLFOXIRI	2	S1	1
	FOLFOX	2	XELOX	8	FOLFOX	3		
	XELOX	23	XELOXIRI	2	Tomudex	1		
	XELOXIRI	1			CPT-11 + S1	1		
	L-OHP + FT207	1						
Mutant	chemotherapy alone	*N* = 3	Plus Bev	*N* = 4	Plus CET	*N* = 1	Plus T-mab	*N* = 1
Combined treatment	FOLFIRI	1	XELOX	4	CPT-11+ S1 + vemurafenib	1	XELOXIRI + pertuzumab	1
	FOLFOX	2						

*WT KRAS/NRAS/BRAF* wild-type, *Bev* bevacizumab, *CET* cetuximab, *T-mab* trastuzumab, *FOLFIRI* Folinic acid, fluorouracil and irinotecan, *FOLFOXIRI* Fluorouracil, folinic acid, oxaliplatin and irinotecan, *FOLFOX* Folinic acid, fluorouracil and oxaliplatin, *L-OHP + FT207* oxaliplatin and 5-fluorouracil

**Table 4:** Treatment response evaluations in patients who received chemotherapy plus trastuzumab

Group (A)	first-line	second-line	third-line	four-line
Number of cases	*N* = 3	*N* = 16	*N* = 9	*N* = 5
CR	0 (0.0%)	0 (0.0%)	0 (0.0%)	0 (0.0%)
PR	1 (51.6%)	4 (25.0%)	0 (0.0%)	0 (0.0%)
SD	2 (42.2%)	10 (62.5%)	6 (66.7%)	2 (40.0%)
PD	0 (0.0%)	2 (12.5%)	3 (33.3%)	3 (60.0%)
ORR	1 (51.6%)	4 (25.0%)	0 (0.0%)	0 (0.0%)
DCR	3 (93.8%)	14 (87.5%)	6 (66.7%)	2 (40.0%)

### Adverse events

According to the Common Terminology Criteria for Adverse Events 3.0 (CTC 3.0), toxic side effects were classified into 1–4 grades. Most of the adverse events in this study were related to chemotherapy drugs. The common toxic side effects of each group are shown in Table 5.

**Table 5:** Toxic side effects in each group of *KRAS/NRAS/BRAF* WT patients (N)

Toxic side effect, Grade	Chemotherapy alone		Plus Bev		Plus CET		Plus T-mab	
	1 ~ 2	3 ~ 4	1 ~ 2	3 ~ 4	1 ~ 2	3 ~ 4	1 ~ 2	3 ~ 4
Myelosuppression	6	3	3	1	2	0	2	1
Gastrointestinal side effects	13	4	2	1	1	1	9	3
Liver dysfunction	3	1	1	0	1	0	2	0
neurotoxicity	1	0	0	0	1	0	0	0
Hand-foot syndrome	1	1	1	0	1	0	0	0
rash	0	0	0	0	3	1	0	0
hemorrhages	1	0	1	0	0	0	0	0
proteinuria	0	0	2	0	0	0	0	0

### Survival analysis

The final follow-up for all patients occurred on 19 February 2021. All 63 patients (100%) fulfilled the follow-up criteria, and 25 patients (39.7%) died during the follow-up period because of the progression of the disease. Male, primary lesions in the right colon, simultaneous metastasis, and unresectable primary lesions may be significant risk factors for poor survival in our univariate analysis (Table 6). When multivariate analysis with Cox regression was performed, we found that the two factors of gender and resection for primary lesion may be independent prognostic factors (Table 7).

**Table 6:** Univariate survival analyses of patients according to various clinicopathological variables

Variable	Median OS(M)	*P*	Variable	Median OS(M)	*P*
Age, Years			Tumor Differentiation		
< 54	48.2	0.923	Poor	31.2	0.193
≥ 54	48.9		Medium	48.2	
Sex			Well	not reached	
Male	31.2	0.02	primary lesion resection		
Female	48.9		Yes	48.2	0.001
Primary site			No	10.2	
Left semi-colon and rectum	48.2	0.006	Metastasectomy		
Right colon	9.4		Yes	32.3	0.415
Number of metastatic organs			No	48.2	
single	49.9	0.485	Status of *KRAS NRAS BRAF*		
≥ 2	41.9		Mutant	32.2	0.085
Metastatic patterns			WT	48.2	
synchronous	32.2	0.012	Adjuvant chemotherapy		
Metachronous	49.9		Yes	48.9	0.227
Therapy Line			No	48.2	
First-line	20	0.223	local therapies		
Second-Line	41.9		Yes	41.9	0.347
Third- or later-line	48.9		No	50.7

**Table 7:** Multivariate Cox model analyses of prognostic factors

Variable	HR	95% CI	*P*
Gender	0.227	0.088 ~ 0.590	0.002
Primary site	3.034	0.810 ~ 11.369	0.100
Metastatic patterns	0.126	0.183 ~ 1.232	0.126
Primary lesion resection	4.835	1.491 ~ 15.684	0.009

**Fig. 1:**
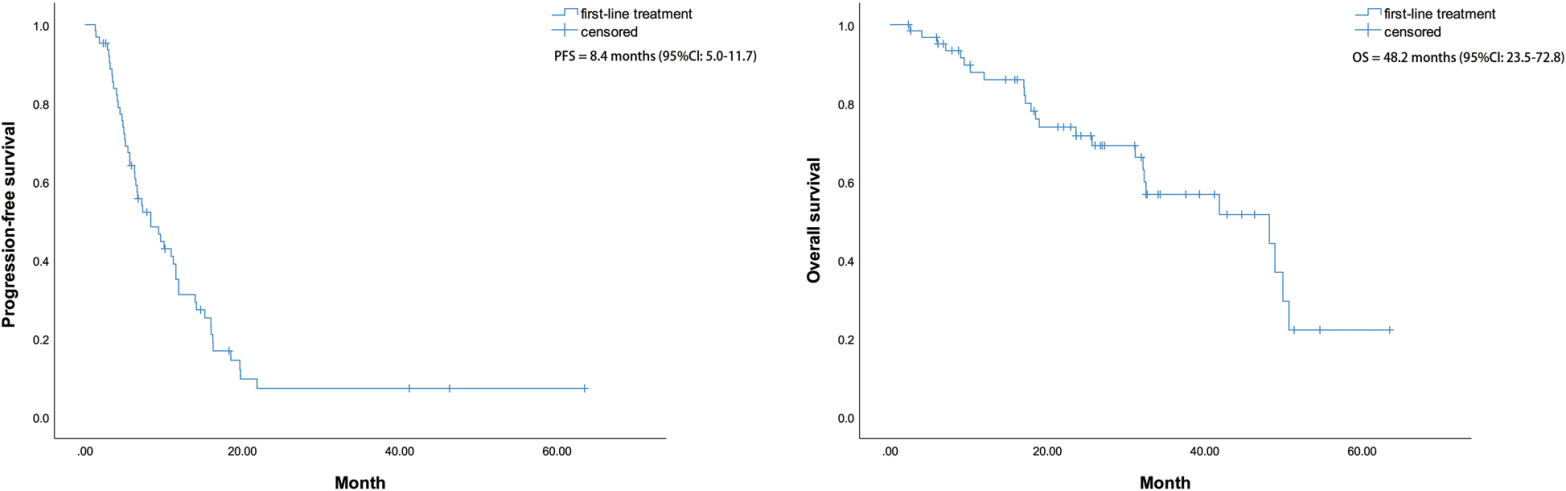
Kaplan–Meier curves of PFS and OS in patients with the first-line treatment

**Fig. 2:**
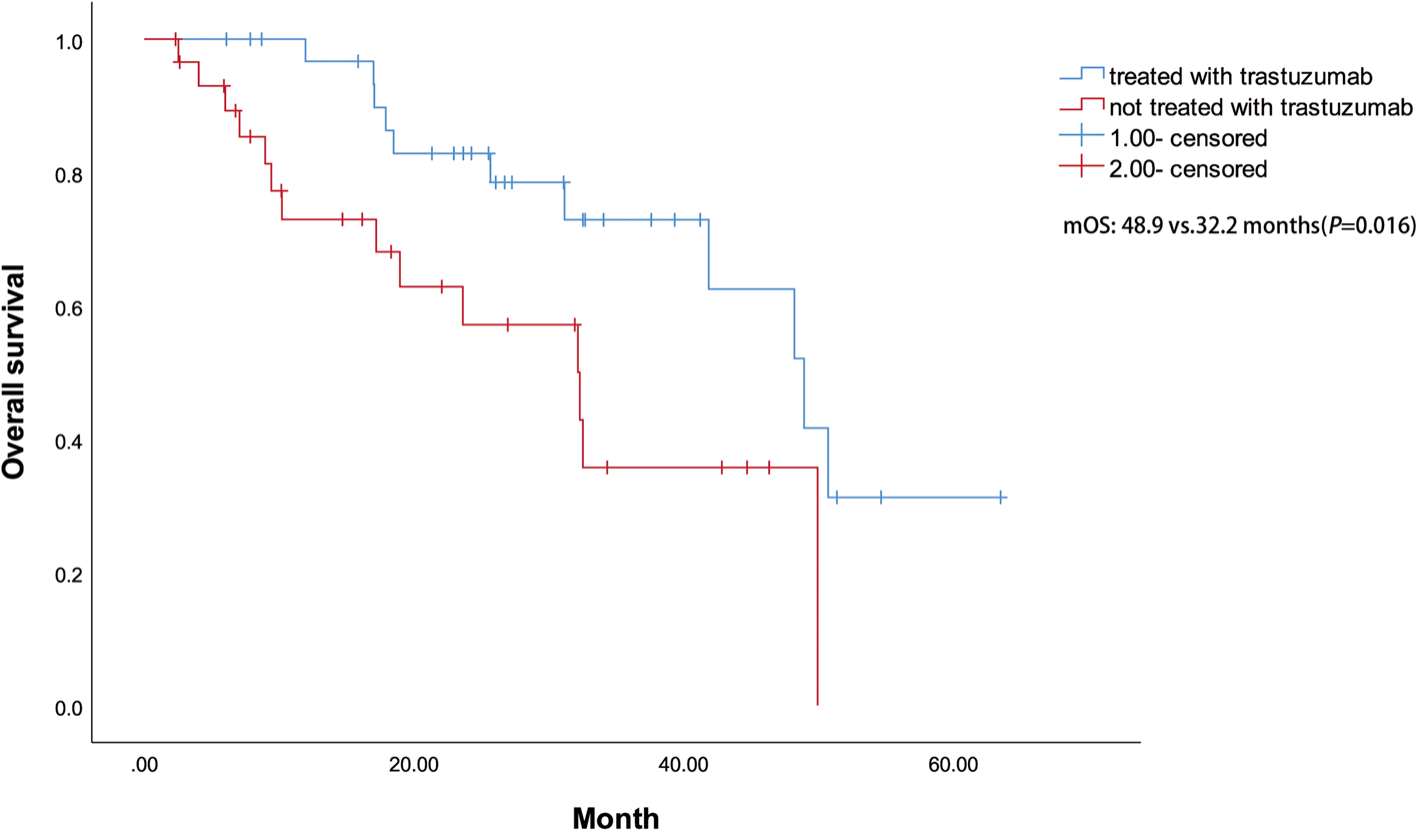
Kaplan–Meier curves of OS in patients who received chemotherapy with or without trastuzumab

**Fig. 3:**
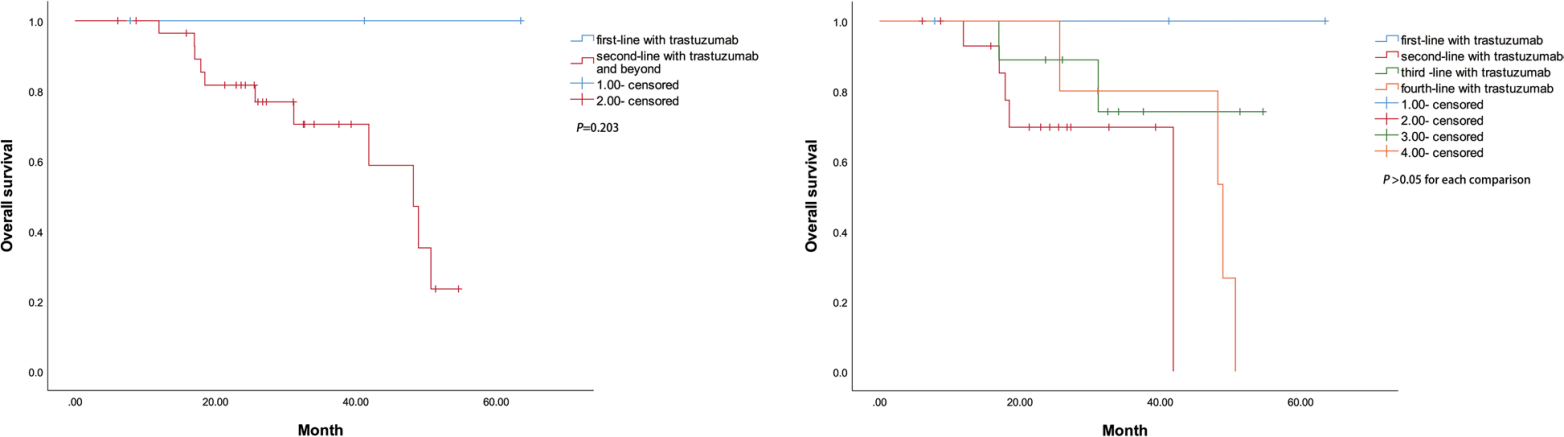
Kaplan–Meier curves of overall survival in patients who received chemotherapy plus trastuzumab at different lines

The median PFS among all 63 patients with the first-line treatment was 8.4 months [95% CI: 5.0–11.7] and the median OS was 48.2 months (95% CI: 23.5–72.8) with the first-line treatment (Fig. 1). The log-rank test revealed a significant difference in median OS survival between group A (treated with trastuzumab) and B (not treated with trastuzumab) (χ^2^ = 5.852, *P* = 0.016) (Fig. 2). There is no significant difference in survival between patients in the first-line treatment group and patients in the second-line and subsequent-line treatment groups (*P* = 0.203). The differences in survival between patients of the first-, second-, third-, and fourth-line treatment groups were not significant. The corresponding Kaplan-Meier analyses are depicted in Fig. 3.

**Fig. 4:**
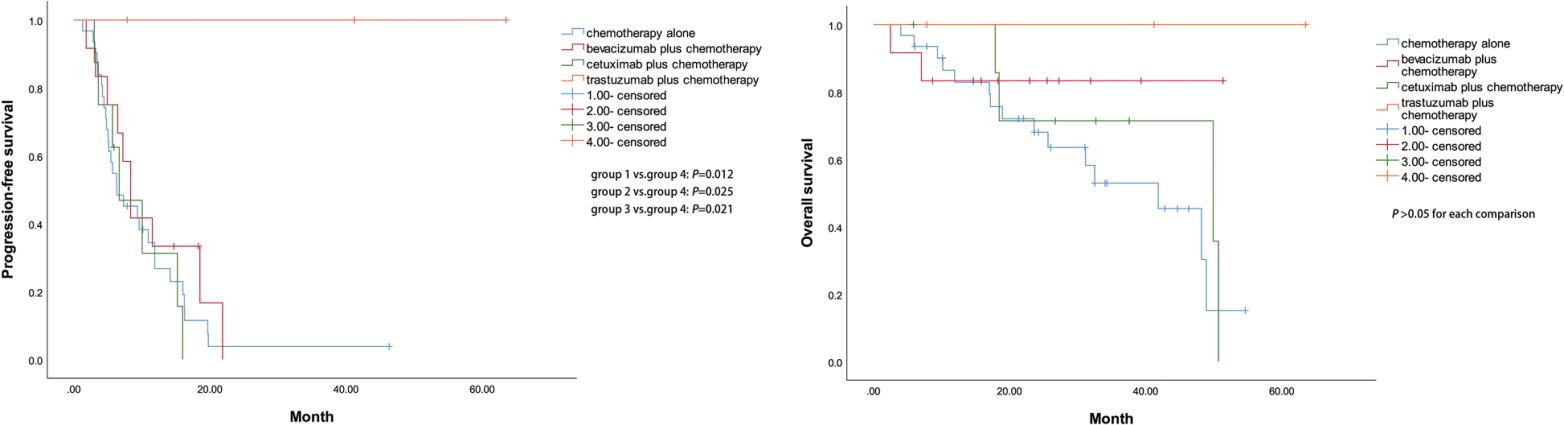
Kaplan–Meier curves of PFS and OS in *KRAS*/*NRAS*/*BRAF* WT patients according to four different first-line therapies

The median PFS time of *KRAS/NRAS/BRAF* WT patients with mCRC treated with four different first-line therapies were 6.3 months (95% CI: 2.1–10.5) in group 1 (chemotherapy alone), 8.4 months (95% CI: 6.5–10.2) in group 2 (bevacizumab plus chemotherapy), 6.7 months (95% CI: 1.6–11.8) in group 3 (cetuximab plus chemotherapy), and PFS was not reached in group 4 (trastuzumab plus chemotherapy). The median OS was 41.9 months (95% CI: 28.4–55.4) in group 1 and 50.0 months (95% CI: 3.9–95.9) in group 3. However, OS in group 2 and group 4 was not reached. The log-rank test revealed a significant difference in median PFS survival between group 1 and group 4 (χ^2^ = 6.308, *P* = 0.012), group 2 and group 4 (χ^2^ = 5.021, *P* = 0.025), group 3 and group 4 (χ^2^ = 5.302, *P* = 0.021), respectively. However, there was no significant difference in the median OS between these four groups (*P* > 0.05 for each comparison). The corresponding Kaplan-Meier analyses are depicted in Fig. 4.

## Discussion

Herein, among 63 HER2-positive mCRC patients, 54 (85.7%) were *KRAS*/*NRAS*/*BRAF* WT. The primary tumor site was left-sided in 88.9% of patients. This suggests that overexpression of HER2 is more likely to occur in patients with left hemicolorectal cancer and *KRAS*/*NRAS*/*BRAF* WT colorectal cancer. Some previous CRC studies have reported that primary location and *KRAS* status are related to the amplification of HER2. A previous study retrospectively assessed the prevalence and putative clinicopathological significance of HER2/neu overexpression/amplification in a series of 1645 CRC samples. HER2/neu-positive tumors tended to be more frequent in colon sigmoideum/ rectum than in the ascending/descending colon (18 vs. 6) [10]. In the HERACLES trial, among 27 patients with HER2-positive mCRC, 23 were rectal (7) and distal colon (16), and 4 were proximal colon cancer [6]. In a meta-analysis of mCRC including 1914 stage II-III patients and 1342 stage IV patients, HER2-overexpression was associated with the WT *KRAS*/*BRAF* status, where 5.2% of cases showed WT *KRAS*/*BRAF* versus only 1.0% containing mutated tumors (*P <* 0.0001) in stage IV and 2.1% versus 0.2% in stage II–III tumors (*P =* 0.01), respectively [7].

In univariate analysis, although the sample size was small, it was found that males, primary lesions of the right colon, simultaneous metastasis and unresectable primary lesions may be important risk factors for low survival. Previous retrospective studies on patients with HER2-positive mCRC are relatively lacking. We will continue to collect data from HER2-positive mCRC patients for analysis and further confirmation of the results.

The outcomes of anti-HER2 agents in mCRC are uncertain during the last decade. Ramanathan et al. reported a phase II study of treatment with trastuzumab combined with irinotecan in nine advanced CRC patients overexpressing HER2. 11 of 138 (8.0%) screened tumors (2+ in 5 and 3+ in 6 patients) detected HER2/neu overexpression via IHC. Among 7 evaluable patients, 5 (71%) achieved a partial response to the treatment with trastuzumab and irinotecan. This study further found that irinotecan did not alter the pharmacokinetic activity and effects of trastuzumab. However, the low rate of HER-2/neu overexpression in advanced CRC patients restricted further investigation and caused the premature termination of the study [11]. From Fig. 2 in this study, it can be seen that for patients with HER2-positive mCRC, patients in the chemotherapy combined with trastuzumab group have better efficacy than those without trastuzumab. This result has potential significance for clinical practice. At the same time, it can be seen from Fig. 4 that in the first-line treatment of *KRAS/NRAS/BRAF* WT patients, chemotherapy combined with trastuzumab seems to be more effective than the regimens of chemotherapy with bevacizumab, chemotherapy with cetuximab, or chemotherapy alone. However, because the sample size of the fourth group is too small, we hope to continue to expand the sample size for further analysis.

In addition, the median PFS of group 3 (cetuximab plus chemotherapy) was shorter compared to that of group 2 (bevacizumab plus chemotherapy) in *KRAS*/*NRAS*/*BRAF* WT mCRC with first-line treatment (6.7 versus 8.4 months). Although not statistically significant, HER2/neu-positive mCRC displayed a tendency to reduce progression-free survival. HER2 amplification as a negative predictor of the response to cetuximab was also proposed in earlier studies [12, 13]. In a retrospective clinical series, Yonesaka et al. evaluated 233 cetuximab-treated patients for the clinical impact of HER2 amplification [14]. Compared to patients without HER2-amplified tumors, those with HER2-amplified tumors exhibited significantly shorter durations for PFS (89 vs. 149 days) and OS (307 vs. 515 days) [14]. Another study analyzing the relationship between HER2 amplification and the efficacy of anti-EGFR monoclonal antibody therapy in *RAS* and *BRAF* WT mCRC patients showed that anti-EGFR agent-treated HER2-positive patients (*n* = 79) showed worsened outcomes compared to patients lacking the anti-EGFR treatment (ORR, 31.2% vs. 46.9%, *P* = 0.031; PFS, 5.7 months vs. 7 months, *P* = 0.087) [15].

In patients with HER2-positive mCRC treated with trastuzumab, although the survival rate of patients in the first-line treatment group was not significantly different from that of patients in the second-line and subsequent treatment groups, However, it can be seen from Fig. 3 that the efficacy of the first-line treatment group has a better trend than that of the second-line and subsequent treatment groups. Due to the small sample size, we hope to expand the sample size in the future to further clarify whether the early application of anti-HER2 drugs will achieve better efficacy.

The prognostic role of HER2 in mCRC remained uncertain until now. In this study, the median PFS of each group was shorter than that reported in previous studies. 1.6% of cases were found to be HER2/neu positive in a previous study that examined 1645 cases of primary colorectal carcinoma [10]. Furthermore, this study revealed that HER2/neu positivity was significantly correlated with advanced lymph node metastases and Union for International Cancer Control (UICC) stages [10]. Concerning long-term implications, HER2/neu-positive colorectal carcinomas are significantly correlated with reduced OS [16]. These results indicated that there may be a negative prognostic association between HER2 amplification and the *KRAS*/*NRAS*/*BRAF* WT status in advanced CRC patients undergoing first-line treatment.

Although the US FDA has not approved any anti-HER2 therapies for the treatment of mCRC so far, the first-generation anti-HER2 molecules such as trastuzumab (Herceptin), lapatinib (Tykerb) and Pertuzumab (Perjeta) Has become the subject of many studies. In preclinical therapeutic trials, Bertotti et al. genetically characterized a large xenograft cohort of mCRC samples (“xenopatients”) to reveal new oncoprotein targets and factors that contribute to therapeutic responses [13]. Patient-derived mCRC xenografts exhibiting HER2 amplification were sensitive to pertuzumab or cetuximab HER2 inhibition in combination with lapatinib, but treatment with pertuzumab alone or in combination with cetuximab had no effect [13]. These data support the development of clinical methods that target HER2 amplification in mCRC. HERACLES-A, a multicentre, open-label, phase II trial, aimed to provide proof of concept for the antitumor activity of trastuzumab and lapatinib in HER2-positive CRC patients [6]. Adult patients possessing the WT *KRAS* exon 2 (codons 12 and 13) and HER2-positive refractory mCRC were enrolled into a standard of care protocol including cetuximab or panitumumab. IHC and FISH were used to verify patient HER2 status using established CRC-specific diagnostic criteria. Of 914 patients with WT *KRAS* exon 2 (codons 12 and 13) mCRC, and 48 patients (5%) with HER2-positive tumors were identified. Among 27 evaluable patients, one, seven, and 12 patients exhibited a complete response, a partial response, and stable disease, respectively. The ORR was 30% and the median OS was 46 weeks. HERACLES-B, a single-arm phase II trial, assessed the efficacy of combined pertuzumab and trastuzumab emtansine (T-DM1) treatment [17]. Among 31 evaluable patients, three and 21 patients exhibited a partial response and disease stabilization, respectively. Finally, the phase IIa multiple basket study MyPathway assessed the activity of a combination of pertuzumab and trastuzumab in HER2-amplified mCRC patients [18]. Among 57 patients with HER2-amplified mCRC, only one patient showed a complete response and 17 had partial responses. Thus, 18 (32%) patients in total achieved a marked response, but further work is needed to develop drugs and drug combinations effective against HER2-amplified mCRC. Dual HER2 inhibition with trastuzumab plus lapatinib or pertuzumab has shown promising preliminary anti-tumoral efficacy in RAS wild-type mCRC. For a limited subgroup (around 5%) of mCRC patients refractory to chemotherapy drugs and anti-EGFR-targeted drugs such as cetuximab and panitumumab, targeting HER2 represents an additional but valuable therapeutic strategy. When applying HER2 blockers in this situation, studies have shown that dual HER2 blockers have advantages [19].

In conclusion, the anti-HER2 targeting drug (trastuzumab) combined with chemotherapy achieved a better therapeutic effect in comparison to other agents in this study, especially in the first and second-line treatment for advanced HER2 positive colorectal cancer. From this retrospective analysis, it can be seen that anti-HER-2 targeted therapy has achieved good results in patients with HER-2 positive colorectal cancer, and it is worthy of further prospective randomized controlled studies.

## Data Availability

All data generated or analysed during this study are included in this published article.
